# Enah overexpression is correlated with poor survival and aggressive phenotype in gastric cancer

**DOI:** 10.1038/s41419-018-1031-x

**Published:** 2018-09-24

**Authors:** Di Chen, Li Xu, Xiaowei Li, Yi Chu, Mingzuo Jiang, Bing Xu, Min Zhao, Weijie Wang, Hua Wang, Huijie Kang, Kai Wang, Kaichun Wu, Jie Liang, Gui Ren

**Affiliations:** 1 State Key Laboratory of Cancer Biology and Xijing Hospital of Digestive Diseases, the Air Force Military Medical University, 127 Changle Western Road, Xi’an, Shaanxi Province 710032 China; 2 Department of Hematology, People’s Liberation Army Center of Hematologic Disorders, Xijing Hospital, the Air Force Military Medical University, 127 Changle Western Road, Xi’an, Shaanxi Province 710032 China; 3 Department of Gastroenterology, the 16th Hospital of the People’s Liberation Army of China, 219 Tuanjie South Road, A Letai, Xinjiang Province, 836599 China; 4 Department of Gastroenterology, Navy General Hospital, Beijing, 100048 China

## Abstract

Enabled homolog (Enah), which is a member of the Ena/VASP family that also includes VASP (vasodilator-stimulated phosphoprotein) and Ena/VASP like, is a mammalian ortholog of Drosophila Enabled (Ena). An increasing number of studies demonstrated Enah overexpression is involved in human colorectal carcinomas, breast cancers and hepatocellular carcinoma. However, the significance of Enah expression in gastric cancer (GC) is poorly elucidated. Here, we demonstrate that Enah is upregulated in GC and associated with AJCC stage, depth of invasion and poor overall survival (OS). Knockdown of Enah inhibited GC cell proliferation and metastasis and vice versa. Further experiments suggested that p-Erk1/2, p-AKT, p-p65, Vimentin and Fibronectin were downregulated and E-cadherin was upregulated after Enah silencing, implicating altered functions in GC proliferation and metastasis. Thus, our study suggests that Enah is a harmful factor for GC and a novel target for GC treatment.

## Introduction

Gastric cancer (GC) is the fourth most common cancer and the second most common cause of cancer-related death^1^. Each year, approximately 990,000 people are diagnosed with GC in the world, of whom about 738,000 die from this disease^2,3^. GC incidence rates differ in sexes and nations. Rates are two to three folds higher in men than women^2^, and the highest incidence rates are observed in East Asia, East Europe, and South America, while the lowest rates in North America and most parts of Africa^4^. In recent years, with the combination of chemotherapy, radiotherapy and surgery treatment, the quality of life of GC patients has improved, but the prognosis of GC patients still makes people feel dissatisfactory. GC was always diagnosed at advanced stage with lymph node metastasis and distant metastasis, one of the most important reasons is lack of early molecular marker. Therefore, it is very important for us to look for better biomarkers to diagnose, guide clinical treatment and predict prognosis for GC.

Enabled homolog (Enah), which is a member of the Ena/VASP family that also includes VASP (vasodilator-stimulated phosphoprotein) and Ena/VASP like, is a mammalian ortholog of Drosophila Enabled (Ena)^5^. The Ena/VASP family plays an important role in regulating cell movement, morpholology and adhesion, processes required during invasion and metastasis^6,7^. Many studies have demonstrated that Enah is dysregulated in many human solid tumors including colorectal carcinomas^8,9^, hepatocellular carcinoma^10^, cervical carcinoma^11^, breast carcinoma^12^, salivary gland carcinoma^13^ and pancreatic carcinoma^14^. However, the potential role of Enah in the development of GC is poorly elucidated.

In this study, we evaluated the expression of Enah through a public database Oncomine, a tissue microarray (TMA), 39 paired GC samples and GC cell lines and analyzed the correlation between Enah expression and clinicopathological parameters and survival of GC patients. Furthermore, we investigated the role of Enah in cell proliferation and metastasis in vitro and in vivo. We also explored three signaling pathways and EMT process that were significantly changed after knockdown and overexpression of Enah.

## Materials and methods

### Human tissue samples and cell lines and cultures

Thirty-nine pairs of human GC tissues and corresponding normal tissues were obtained from the Department of Digestive Diseases, Xijing Hospital. The ethics committee at Xijing Hospital of the Fourth Military Medical University approved this study, and all the patients gave written informed consent on the use of clinical specimens for medical research. The human GC cell lines (MKN28, MKN45, SGC7901, BGC823 and AGS) and the immortalized gastric epithelial cell line GES were preserved at our institute. All the cell lines were cultured in RPMI-1640 medium (GIBCO) supplemented with 10% foetal bovine serum (GIBCO) and 100 U/ml penicillin/streptomycin and cultured at 37 °C in a humidified incubator containing 5% CO_2_.

### Quantitative real-time polymerase chain reaction (qRT-PCR)

Total RNA from human GC cell lines, GC tissues and their adjacent normal tissues were extracted using TaKaRa MiniBEST Universal Extraction Kit (TaKaRa) according to the manufacturer’s instructions and cDNA was synthesized using PrimeScript^TM^ RT Master Mix (Perfect Real Time) (TaKaRa). The reaction system (10 ul) contains 5x PrimeScript^TM^ RT Master Mix, Total RNA and RNase Free dH2O. For PCR, the reaction system (20 ul) consists of SYBR Premix Ex Taq^II^ 10 ul, PCR Forward Primer (10 uM) 1 ul, PCR Reverse Primer (10 uM) 1 ul, cDNA 2 ul and sterile distilled water (dH_2_O) 6 ul. The primer sequences were as follows: Enah (sense: 5′-GTGGCTCAACTGGATTCAGCA-3′, antisense 5′-AGGAATGGCACAGTTTATCACGA-3′);β-actin (sense 5′-TGGCACCCAGCACAATGAA-3′, antisense 5′- CTAAGTCATAGTCCGCCTAGAAGCA-3′). The relative quantitation of gene expression levels were determined by the 2^−△△^Ct method and β-actin was used as a reference.

### Western blot

Total proteins were extracted with lysis buffer (RIPA, protease inhibitor and phosphatase inhibitor) from the cultured cells. The proteins were separated by SDS-PAGE and transferred to nitrocellulose membrane. The membranes were blocked with 5% milk and then incubated with primary antibodies at 4 °C overnight. The primary antibodies used in the experiment were as following: Enah (BD Bioscience; US); PCNA (Cell Signaling Technology, USA), p-Erk1/2/Erk1/2 (Cell Signaling Technology, USA), p-STAT/STAT (Cell Signaling Technology, USA), p-AKT/AKT (Cell Signaling Technology, USA), p-p65/p65 NF-κB (Cell Signaling Technology, USA), E-cadherin (Cell Signaling Technology, USA),Vimentin (Abcam, USA), Fibronectin (Santa Cruz Biotechnology, USA) and β-actin (Sigma, USA). After washing three times with 1 × TBST, the membranes were incubated with horseradish peroxidase (HRP)-conjugated goat anti-mouse or anti-rabbit IgG (Zhong Shan Goldenbridge Biotech, China). At last, the membranes were visualized using Immobilon^TM^ Western Chemiluminescent HRP Substrate (Millipore).

### Lentiviral transfection

All the recombinant lentiviruses were purchased from Genechem Corporation (Shanghai, China). Lentiviral vectors encoding shRNAs were designated LV-shEnah and LV-shcontrol. Lentiviral vector encoding the human Enah gene was designated LV-Enah and an empty vector which was used as the negative control was designated NC. Lentiviral transfection was performed according to the protocol of the Genechem Recombinant Lentivirus Operation Manual provided by Genechem Corporation. The transfection efficiencies were determined by examining GFP expression under a microscope and the expression of Enah was measured using qRT-PCR and Western blot analysis.

### 5-Bromo-2-deoxyUridine (BrdU) incorporating assay and colony forming assay

The BrdU incorporating assay was performed using BrdU Cell Proliferation Assay Kit (Millipore, USA) according to the manufacturer’s instructions. For colony forming assay, 1000 cells were seeded in six-well plates and cultured at 37 °C in a humidified incubator. After two weeks’ incubation, the cells were fixed with 4% paraformaldehyde, then stained with 0.5% gentian violet and observed under a digital camera.

### Cell cycle and apoptosis analysis

Cell cycle and apoptosis were measured using a FACScan flow cytometer (BD Biosciences, Franklin Lakes, NJ). For cell cycle analysis, the cells were starved in serum-free medium for 24 h, then cultured normally for less than 24 h and harvested with tyrisin (approximately 1 × 10^6^ cells), finally fixed with 75% ethanol and stained with propidium iodide (Sigma, MO). The Annexin V PE/7AAD Kit (Multi sciences) was used for apoptosis assay. The apoptosis of cells was induced with 5-FU (1 mg/ml) and stained according to the manufacturer’s protocol. The results were analyzed using Multicycle-DNA Cell Cycle Analyzed Software.

### High-content cell mobility assay

We performed high-content assays to assess cell motility and the protocol referred to the “Material and methods” section of a previous report^15^.

### Transwell assay

The transwell assay was performed using 6.5-mm transwell chambers with a pore size of 8 μm (Corning Costar Corp., USA) as described previously^15^. All the assays were performed in triplicate.

### Immunohistochemistry

Paraffin-embedded slides and TMA were dewaxed with dimethylbenzene (I and II) and gradient alcohol (100, 95, 85, 75%), and antigen retrieval was carried out by high-pressure boiler in citrate buffer for 2 min. Slides were incubated with 3% hydrogen peroxide (H_2_O_2_) for 20 min, followed by incubation with goat serum for 30 min at room temperature. Never wash the slides with phosphate-buffered saline, just pour the goat serum on the slides. Then slides were incubated with primary antibodies at 4 °C overnight. Biotinylated secondary anti-rabbit or anti-rat antibodies were added and incubated for 1 h at room temperature. After incubating with streptavidin-HRP for 30 min, slides were stained with DAB (DAB chromogen: DAB substrate = 1:20) and counterstained with haematoxylin. After washing in running water, slides were dehydrated with gradient alcohol and dimethylbenzene. Finally, coverslips were used to mount onto the slides with neutral balsam.

The degree of immunostaining was observed and scored separately by two independent pathologists. Immunoreactivities were scored by the percentage of stained cells (0, no staining; 1, 0~25%; 2, 25~75%; 3, 75~100%) and the intensity of staining (0, no staining; 1, weak = light yellow; 2, moderate = yellow brown; 3,strong = brown).Then a final score ranging from 0 and 9 could be calculated by multiplication of two values and for the purposes of statistical analysis, all cases were grouped into four categories as negative (0~1;−), weak (2; +), moderate (3~5; ++), and strong (6–9; +++). At last, we defined ‘negative and weak expression’ and “moderate and strong expression” as “Enah low expression’ and ‘Enah high expression”, respectively.

### Immunofluorescence staining

We performed immunofluorescence staining to measure the expression of EMT-related markers and the protocol referred to the “Material and methods” section of a previous report^16^.

### Statistical analysis

Paired *t* test was used to analyze the differences of Enah expression in GC cell lines, GC tissues and corresponding adjacent normal tissues. Enah positive rates in GC tissues and matched adjacent normal tissues were compared using chi-square test. The relationship between Enah expression and clinicopathologic parameters was also assessed using chi-square test. Kaplan–Meier method was used to analyze OS of patients and comparisons were analyzed by log-rank test. Cox regression model was used for univariate and multivariate analysis, hazard ratios and their 95% confidence intervals were calculated. All data were analyzed using the Statistical Software Package for the Social Sciences (SPSS; Chicago, IL, USA). All statistical tests were two sided, and *p* < 0.05 was considered statistically significant.

## Results

### Enah is upregulated in GC tissues and cell lines and is correlated with clinicopathological parameters and survival of GC patients

**Fig. 1:**
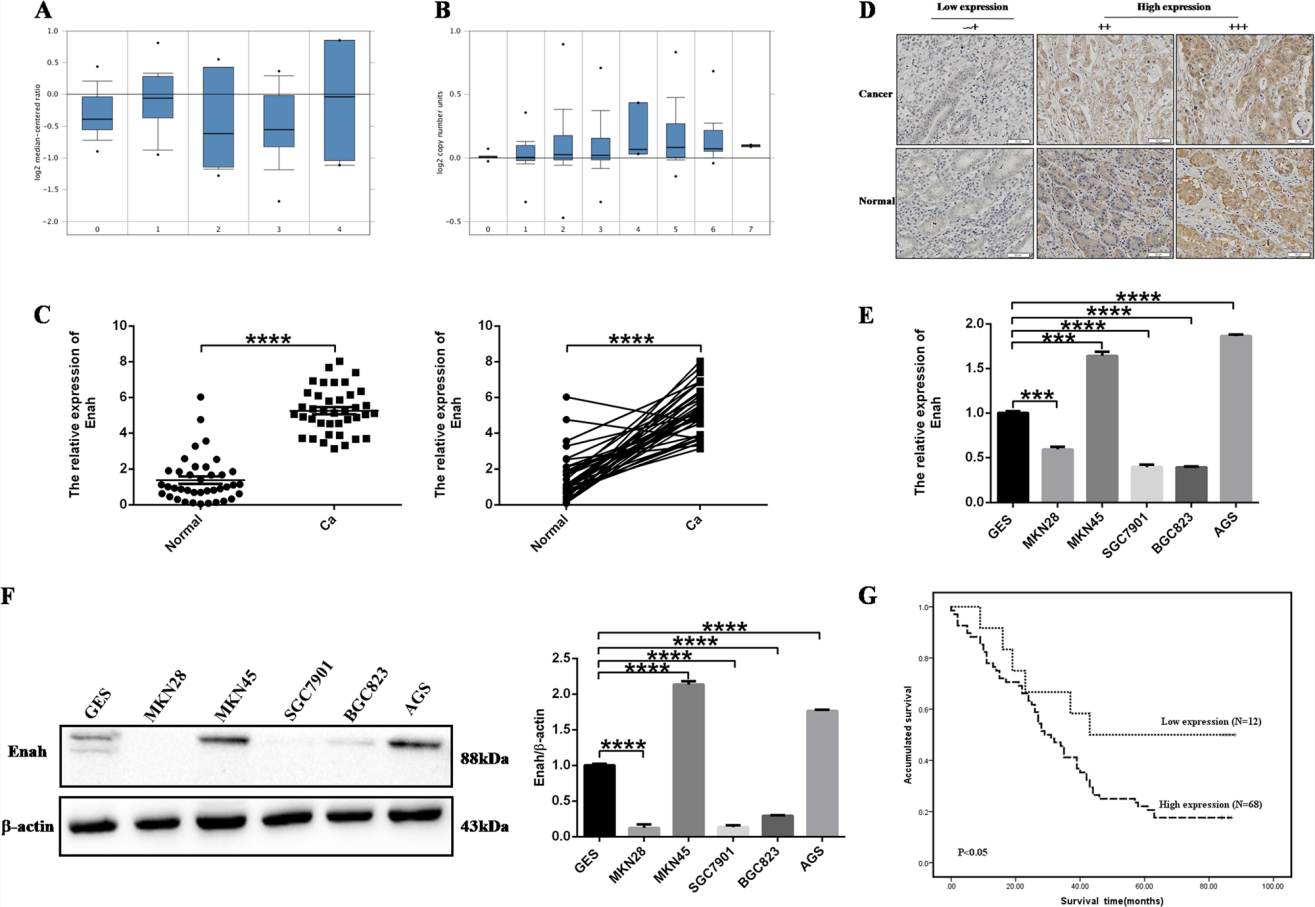
Expression of Enah in GC tissues and cell lines. **a** Enah expression at mRNA level in the Chen Gastric dataset grouped by no value (0), diffuse gastric adenocarcinoma (1), gastric adenocarcinoma (2), gastric intestinal type adenocarcinoma (3) and gastric mixed adenocarcinoma (4). **b** Enah copy number in TCGA Gastric dataset grouped by no value (0), diffuse gastric adenocarcinoma (1), gastric adenocarcinoma (2), gastric intestinal type adenocarcinoma (3), gastric papillary adenocarcinoma (4), gastric tubular adenocarcinoma (5), mucinous gastric adenocarcinoma (6) and signet ring cell gastric adenocarcinoma (7). **c** Expression of Enah in thirty-nine paired GC tisssues and adjacent normal tissues measured by qRT-PCR (left panel: combined comparison; right panel: intra-patient comparison; ****p < 0.0001). **d** Representative immunohistochemical staining of Enah in GC tissues and corresponding normal tissues (Scale: 50 um). **e** Expression of Enah in five GC cell line at mRNA level measured by qRT-PCR (****p < 0.0001). **f** Expression of Enah in five GC cell line at protein level measured by western blot (left panel) and semiquantitative analysis (right panel), with β-actin used as the loading control. **g** Kaplan–Meier Survival Analysis shows High expression level of Enah was associated with worse OS of GC patients (*n* = 80; Low expression: −~+; High expression: ++~+++; *p* < 0.05, log rank test)

To determine the role of Enah in GC, firstly, the Oncomine database was queried and the results showed that Enah mRNA expression was higher in GC tissues compared with corresponding normal tissues (Fig. 1a) and Enah copy number was also increased in GC tissues (Fig. 1b).Then, we obtained thirty-nine paired GC tissues and corresponding normal tissues for qRT-PCR and as shown in Fig. 1c, in comparison with normal tissues, the expression level of Enah mRNA is significantly higher in GC tissues. Furthermore, we measured the Enah expression through immunohistochemistry with a TMA which contained ninety paired GC tissues and adjacent normal tissues. We found that Enah positive rate was 84.44% (76/90) in GC tissues and 51.11% (46/90) in non-cancerous adjacent tissues with a significantly statistical difference (*p* < 0.001, Table 1) and Enah was mainly present in the cytoplasm of tumor cells (Fig. 1d). Finally, we measured the expression of Enah in five GC cell lines, and the results showed that Enah was up-regulated in two GC cell lines MKN45 and AGS compared with GES at not only RNA level (Fig. 1e) but also protein level (Fig. 1f). Collectively, these data demonstrated that Enah was increased in GC.

**Table 1:** The expression level of Enah in GC tissues and adjacent normal tissues (*n* = 90)

Tissue types	Total	Enah expression			*p*
		Negative (−~+)	Positive (++)	Strong positive (+++)	
Gastric cancer	90	14	23	53	<0.001
Normal tissue	90	44	38	8	

In order to understand the potential roles of Enah in the development of GC, we analyzed the relationship between Enah expression and clinicopathological characteristics. The results showed Enah expression was significantly associated with AJCC stage (*p* = 0.040) and depth of invasion (*p* < 0.001). However, no significant association was found between Enah expression and other clinicopathological features, such as gender, age, nodal status, distant metastasis and pathological classification (Table 2).

**Table 2:** The relationship between Enah expression and clinicopathological characteristics of GC

Variable		Total	Enah expression			*p*
			Negative (−~+)	Positive (++)	Strong positive (+++)	
		90	14	23	53	
Gender	Male	70	10	21	39	0.192
	Female	20	4	2	14	
Age (y)	≥60	61	8	13	40	0.174
	<60	29	6	10	13	
AJCC stage	Stage 1 + 2	37	10	8	19	0.040
	Stage 3 + 4	53	4	15	34	
Depth of invasion	T1 + T2	11	6	1	4	<0.001
	T3 + T4	79	8	22	49	
Nodal status	N0	23	4	8	11	0.419
	N1 + N2 + N3	67	10	15	42	
Distant metastasis	M0	86	14	21	51	0.430
	M1	4	0	2	2	
Pathological classification	I + II	24	5	5	14	0.646
	III + IV	66	9	18	39	

*AJCC* American Joint Committee on Cancer

To identify whether Enah expression had an effect on the OS of GC patients, we performed Kaplan-Meier survival analysis on those patients with complete survival information (80 cases). The patients with high Enah expression had lower OS rate than those with low expression (*p* < 0.05, Log-rank test, Fig. 1g), with a 5-year OS rate of 22.1%, whereas that in patients with low expression level was 50.0%. Furthermore, we performed univariate and multivariate Cox regression analysis to examine whether Enah expression was an independent prognostic factor for GC patients. In univariate Cox regression analysis, the variables gender, age, depth of invasion, nodal status, distant metastasis, pathological classification and Enah expression were included. Among these parameters, different depth of invasion, nodal status, AJCC stage and Enah expression had significantly statistical differences in survival with *p*-values less than 0.05 (Table 3). In multivariate Cox regression analysis, only one factor, depth of invasion was still significantly different between groups with *p*-values less than 0.05 (Table 4). These results demonstrated that Enah expression may not be identified as an independent predictor for GC patients.

**Table 3:** Univariate analysis of prognostic markers in GC patients (*N* = 80)

Variable	HR (95% CI)	*p*
Gender	0.815(0.448–1.481)	0.501
Male		
Female		
Age (y)	0.863 (0.502–1.481)	0.592
≥60		
<60		
AJCC stage	0.371 (0.213–0.647)	0.001
Stage 1 + 2		
Stage 3 + 4		
Depth of invasion	0.123(0.030–0.506)	0.004
T1 + T2		
T3 + T4		
Nodal status	0.362 (0.183–0.717)	0.004
N0		
N1 + N2 + N3		
Distant metastasis	0.533 (0.192–1.482)	0.228
M0		
M1		
Pathological classification	0.846(0.466–1.535)	0.582
I + II		
III + IV		
Enah	0.596 (0.360–0.988)	0.045
Low expression (−~+)		
High expression(++~+++)		

*HR* hazard ratio, *CI* confidence interval

**Table 4:** Multivariate analysis of prognostic markers in GC patients (*N* = 80)

Variable	HR (95% CI)	*p*
Gender	0.619 (0.316–1.209)	0.160
Male		
Female		
Age (y)	1.159 (0.657–2.047)	0.610
≥60		
<60		
AJCC stage	0.659 (0.357–1.218)	0.183
Stage 1 + 2		
Stage 3 + 4		
Depth of invasion	0.179(0.042–0.769)	0.021
T1 + T2		
T3 + T4		
Nodal status	0.687 (0.262–1.802)	0.445
N0		
N1 + N2 + N3		
Distant metastasis	0.789(0.273–2.279)	0.662
M0		
M1		
Pathological classification	1.031 (0.556–1.913)	0.923
I + II		
III + IV		
Enah	0.684 (0.407–1.151)	0.153
Low expression (**−**~+)		
High expression(++~+++)		

### Reduction of Enah expression inhibits GC cell proliferation and metastasis in vitro

**Fig. 2:**
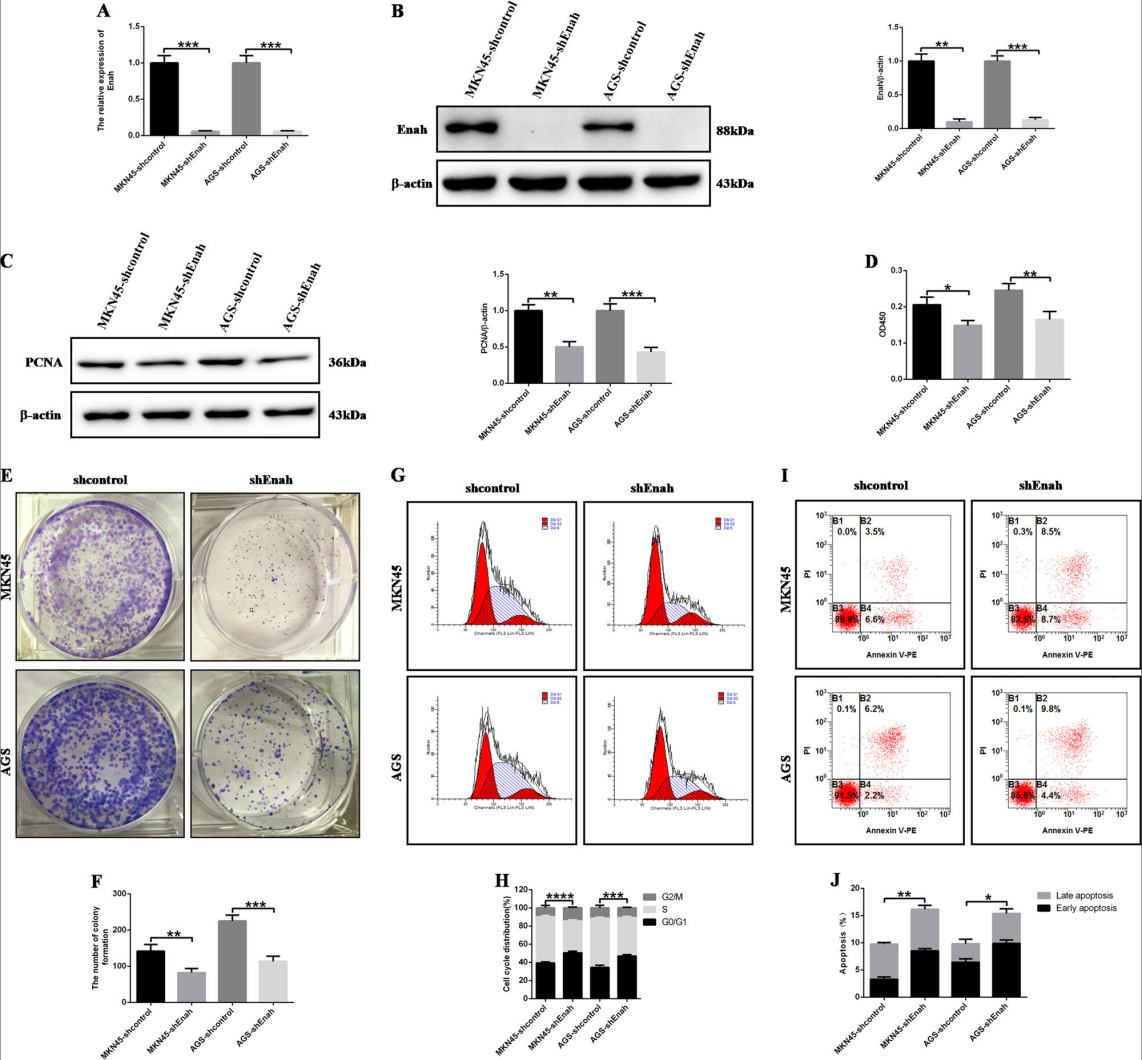
Reduction of Enah expression inhibits GC cell proliferation and promotes cell apoptosis in vitro. **a** Decreased mRNA level of Enah in LV-shEnah cells compared with LV-shcontrol cells. **b** Decreased protein level of Enah in LV-shEnah cells compared with LV-shcontrol cells (left panel) and semiquantitative analysis (right panel). **c** Decreased protein level of PCNA in LV-shEnah cells compared with LV-shcontrol cells (left panel) and semiquantitative analysis (right panel). **d** BrdU assay showed that less BrdU were incorporated in LV-shEnah cells compared with LV-shcontrol cells. **e**, **f** Colony forming assay showed decreased colony formation and colony cell number in LV-shEnah cells compared with LV-shcontrol cells. Representative images, magnification × 200 (**e**) and statistical analysis (**f**). **g**, **h** Cell cycle assays showed more cells arrested in the G0/G1 stages in LV-shEnah cells compared with LV-shcontrol cells. Representative images (**g**) and statistical analysis (**h**). **i**, **j** Apoptosis assays revealed an increased percentage of apoptotic GC cells in LV-shEnah cells compared with LV-shcontrol cells. Representative images (**i**) and statistical analysis (**j**). All the data are expressed as the means ± SD of three independent experiments. *p < 0.05; **p < 0.01; ***p < 0.001; ****p < 0.0001; LV-shEnah versus LV-shcontrol of MKN45 and AGS cells, respectively

**Fig. 3:**
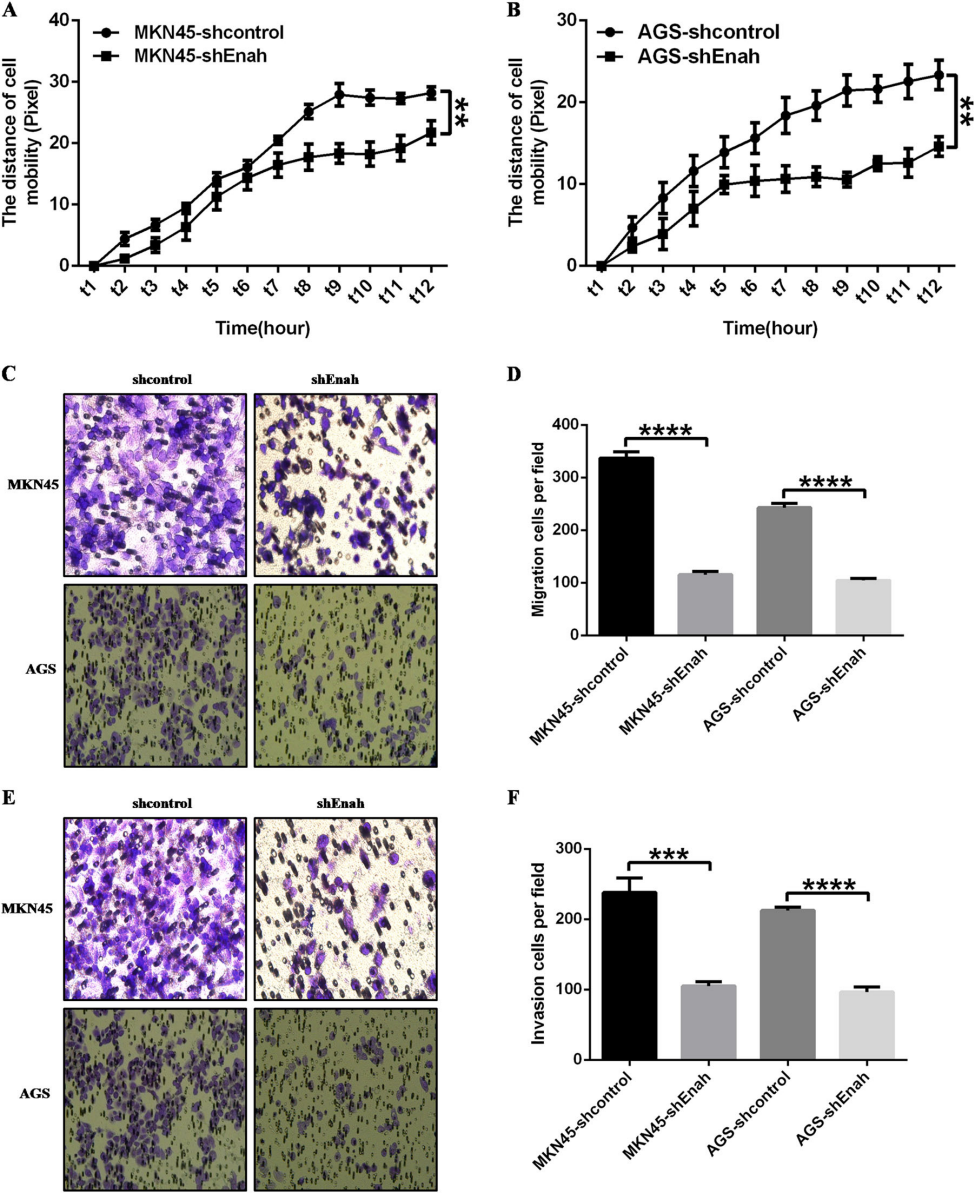
Reduction of Enah expression inhibits GC cell metastasis in vitro. **a**, **b** High-content cell mobility assay indicated impaired mobility in LV-shEnah cells compared with LV-shcontrol cells. MKN45 cell (**a**) and AGS cell (**b**). (C~F) Transwell assay showed the reduced ability of cell migration and invasion in LV-shEnah cells compared with LV-shcontrol cells. Representative photographs of migrating MKN45 and AGS cells, magnification × 200 (**c**) and statistical analysis of the relative numbers of migrating MKN45 and AGS cells (**d**); representative photographs of invading MKN45 and AGS cells, magnification × 200 (**e**) and statistical analysis of the relative numbers of invading MKN45 and AGS cells (**f**). All the data are expressed as the means ± SD of three independent experiments. *p < 0.05; **p < 0.01; ***p < 0.001; ****p < 0.0001; LV-shEnah versus LV-shcontrol of MKN45 and AGS cells, respectively

In order to determine the role of Enah in the GC cell proliferation and migration, we firstly established four stable cell lines (denoted MKN45-shcontrol, MKN45-shEnah, AGS-shcontrol and AGS-shEnah) after transfecting with the LV-shcontrol or LV-shEnah lentivirus, respectively, and the expression level of Enah was detected by PCR and WB. The expression of Enah was decreased compared with the control cells (Figs. 2a, b). PCNA (proliferating cell nuclear antigen), the cell-proliferation marker, was downregulated after Enah knockdown (Fig. 2c). BrdU assay showed that downregulation of Enah expression decreased the BrdU incorporation (Fig. 2d).Colony forming assay showed that reduced Enah level inhibited colony formation and colony cell number (Figs. 2e, f). Cell cycle assays showed that silencing Enah increased the G0/G1 population compared with control cells (Figs. 2g, h). Apoptosis assays revealed that Enah inhibition led to an increased percentage of apoptotic GC cells (Fig. 2i, j). Compared with control cells, MKN45 and AGS cells showed dramatically impaired mobility after Enah knockdown, which was confirmed by high-content cell mobility assay (Figs. 3a, b). Transwell assay showed that downregulation of Enah expression significantly inhibited the migration and invasion capacities of the MKN45 and AGS cells (Figs. 3c~f). Collectively, these data demonstrated that Enah silencing played an inhibitory role in the GC cell proliferation and metastasis.

### Enah knockdown inhibits GC cell proliferation and metastasis in vivo

**Fig. 4:**
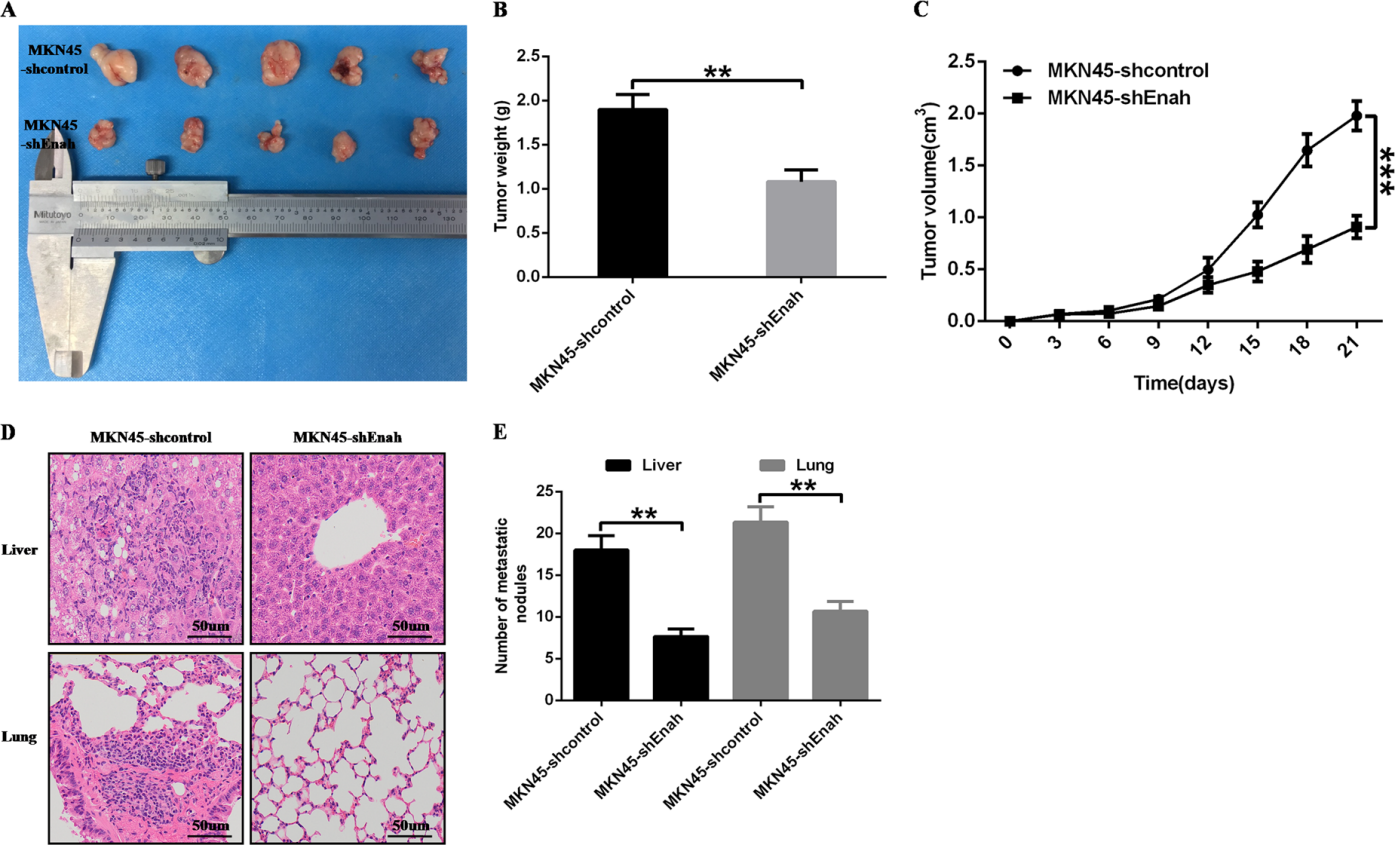
Enah downregulation inhibits GC cell proliferation and metastasis in vivo. **a**~**c** The results of subcutaneously implanted tumor model showed that the xenograft showed lower weight and volume of MKN45-shEnah cells compared with MKN45-shcontrol cells. Representative images of tumors formed in nude mice (*n* = 5) (**a**); quantification of tumor weights of xenograft in mice (**b**); quantification of tumor growth curves of xenograft in mice (**c**). **d**~**e** The results of tail-vein metastasis model showed that the mice formed less tumor nodules in the liver and lung of MKN45-shEnah cells compared with MKN45-shcontrol cells. Representative images showing HE staining of liver and lung tissues isolated from mice injected with MKN45-shEnah cells or MKN45-shcontrol cells (scale: 50 um) (**d**); quantification of the average number of metastatic tumor nodules in the liver and lung of mice (**e**). **p < 0.01; ***p < 0.001; MKN45-shEnah versus MKN45-shcontrol cells

To further investigate the role of Enah in GC cell proliferation and metastasis in vivo, we performed subcutaneously implanted tumor model and tail-vein metastasis model with MKN45-shcontrol and MKN45-shEnah cells. Compared with control group, the xenograft showed lower weight and volume of MKN45 cell that had decreased expression of Enah (Figs. 4a~c). Moreover, the mice formed less tumor nodules in the liver and lung of Enah knockdown group in comparation with control group (Figs. 4d~e).

### Overexpression of Enah promotes GC cell proliferation and metastasis in vitro and in vivo

**Fig. 5:**
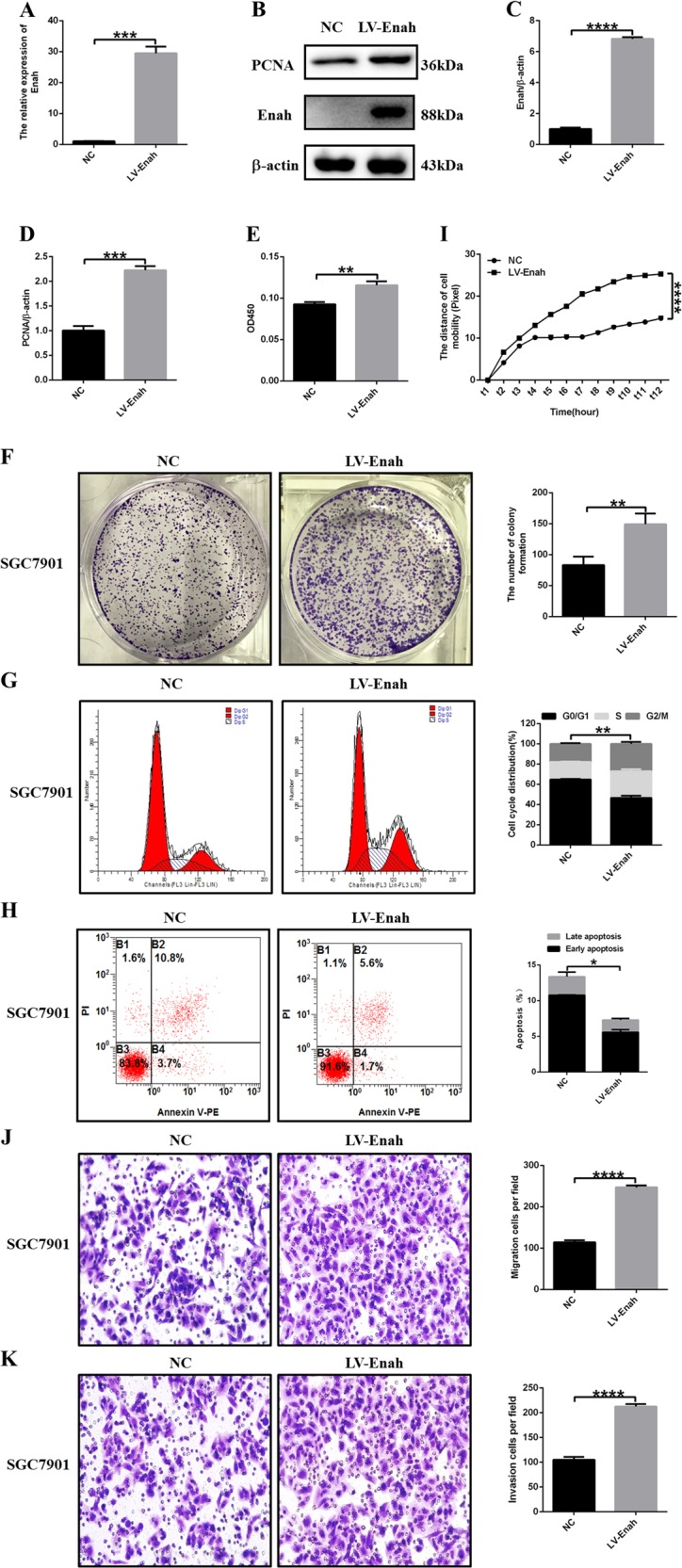
Overexpression of Enah promotes GC cell proliferation, migration and inhibits cell apoptosis in vitro. **a** Increased mRNA level of Enah in LV-Enah cells compared with NC cells. **b** Increased protein level of Enah and PCNA in LV-Enah cells compared with NC cells. **c** The semiquantitative analysis of increased protein level of Enah. **d** The semiquantitative analysis of increased protein level of PCNA. **e** BrdU assay showed that more BrdU were incorporated in LV-Enah cells compared with NC cells. **f** Colony forming assay showed increased colony formation and colony cell number in LV-Enah cells compared with NC cells. Representative images, magnification × 200 (left panel) and statistical analysis (right panel). **g** Cell cycle assays showed less cells arrested in the G0/G1 stages in LV-Enah cells compared with NC cells. Representative images (left panel) and statistical analysis (right panel). **h** Apoptosis assays revealed a decreased percentage of apoptotic GC cells in LV-Enah cells compared with NC cells. Representative images (left panel) and statistical analysis (right panel). **i** High-content cell mobility assay indicated enhanced mobility in LV-Enah cells compared with NC cells. **j** Transwell assay showed the enhanced ability of cell migration in LV-Enah cells compared with NC cells. Representative photographs of migrating SGC7901 cells, magnification × 200 (left panel) and statistical analysis of the relative numbers of migrating SGC7901 cells (right panel). **k** Transwell assay showed the enhanced ability of cell invasion in LV-Enah cells compared with NC cells. Representative photographs of invading SGC7901 cells, magnification × 200 (left panel) and statistical analysis of the relative numbers of invading SGC7901 cells (right panel). All the data are expressed as the means ± SD of three independent experiments. *p < 0.05; **p < 0.01; ***p < 0.001; ****p < 0.0001

To further confirm the stimulative function of Enah in GC development, we upregulated Enah expression with lentivirus using the GC cell line SGC7901. After Enah stable transfected cell line (LV-Enah, SGC7901) was established, PCR and WB were used to detect the expression level of Enah. The expression of Enah in the stable cell line was increased compared with the control cell (NC) (Figs. 5a~c). PCNA was upregulated after overexpression of Enah (Figs. 5b, d). BrdU assay showed that upregulation of Enah expression increased the BrdU incorporation (Fig. 5e).Colony forming assay showed that increased Enah level promoted colony formation and colony cell number (Fig. 5f). Cell cycle assays showed that Enah overexpression decreased the G0/G1 population compared with control cells (Fig. 5g). Apoptosis assays revealed that Enah overexpression led to a decreased percentage of apoptotic GC cells (Fig. 5h). Compared with control cells, SGC7901 cell showed dramatically enhanced mobility after Enah upregulation, which was confirmed by high-content cell mobility assay (Fig. 5i). Transwell assay showed that upregulation of Enah expression significantly promoted the migration and invasion capacities of SGC7901 cell (Figs. 5j, k). Collectively, these data demonstrated that Enah overexpresion played a stimulative role in the GC cell proliferation and metastasis.

**Fig. 6:**
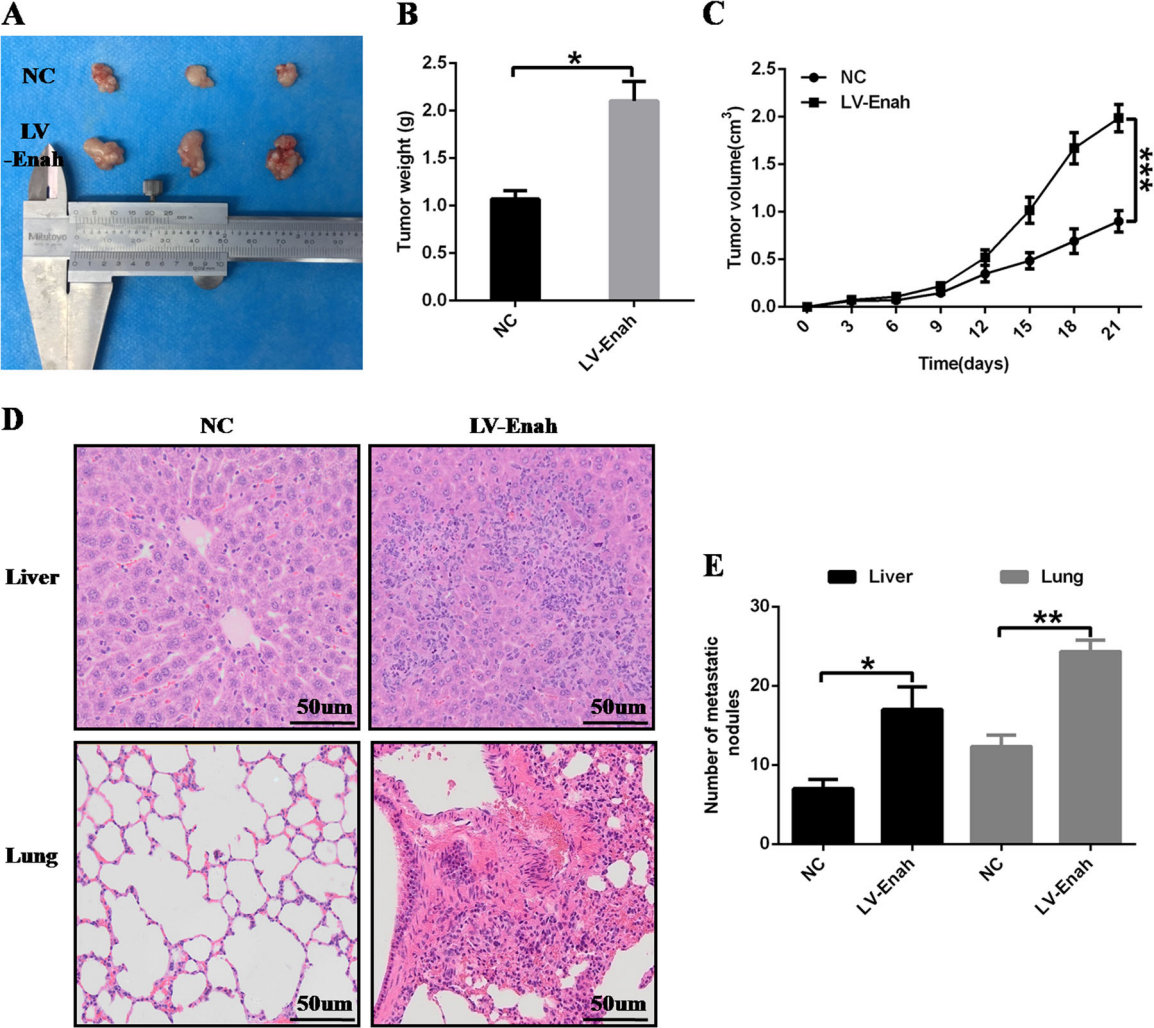
Overexpression of Enah promotes GC cell proliferation and metastasis in vivo. **a**~**c** The results of subcutaneously implanted tumor model showed that the xenograft showed heavier weight and bigger volume of SGC7901 LV-Enah cells compared with NC cells. Representative images of tumors formed in nude mice (*n* = 3) (**a**); quantification of tumor weights of xenograft in mice (**b**); quantification of tumor growth curves of xenograft in mice (**c**). **d**~**e** The results of tail-vein metastasis model showed that the mice formed more tumor nodules in the liver and lung of SGC7901 LV-Enah cells compared with NC cells. Representative images showing HE staining of liver and lung tissues isolated from mice injected with SGC7901 LV-Enah cells or NC cells (scale:50um) (**d**); quantification of the average number of metastatic tumor nodules in the liver and lung of mice (**e**). *p < 0.05; **p < 0.01; ***p < 0.001

In addition, functional assay in vivo also demonstrated that compared with control group, the xenograft showed heavier weight and bigger volume of SGC7901 cell that had increased expression of Enah (Figs. 6a~c). Moreover, the mice formed more tumor nodules in the liver and lung of SGC7901 cell that had increased expression of Enah compared with control group (Figs. 6d~e).

### Reduction of Enah downregulates p-Erk1/2, p-AKT and p-p65 and inhibits EMT (epithelial-mesenchymal transition) progress in GC cells

**Fig. 7:**
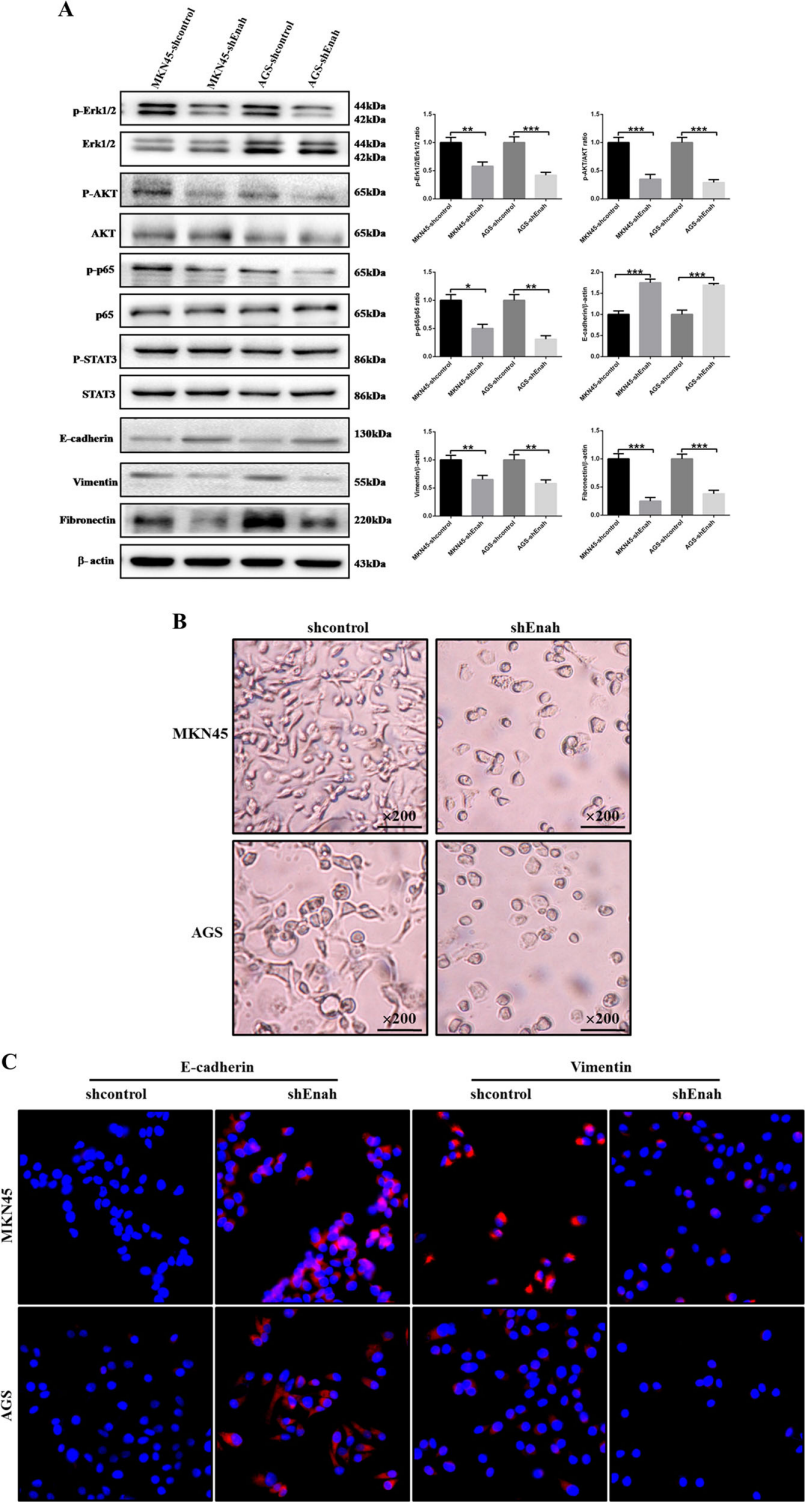
Reduction of Enah downregulates p-Erk1/2, p-AKT, and p-p65 and inhibits EMT (epithelial-mesenchymal transition) progress in GC cells. **a** Decreased protein levels of p-Erk1/2, p-AKT, p-p65, Vimentin, Fibronectin, and increased protein level of E-cadherin in LV-shEnah cells compared with LV-shcontrol cells. No significant difference was detected in the protein expression of p-STAT3. **b** Morphology of MKN45-shcontrol, MKN45-shEnah, AGS-shcontrol and AGS-shEnah cells as visualized using phase-contrast microscopy (magnification × 200). **c** Immunofluorescence analysis of E-cadherin and Vimentin expression in MKN45-shcontrol, MKN45-shEnah, AGS-shcontrol and AGS-shEnah cells. *p < 0.05; **p < 0.01; ***p < 0.001; LV-shEnah versus LV-shcontrol of MKN45 and AGS cells, respectively

Although the role of Enah has been investigated in some tumors, the specific pathway by which Enah functions remains unclear. In order to elucidate the pathway through which Enah regulates cell proliferation and metastasis in GC, we detected protein expression of several canonical signaling pathway correlated with cell proliferation and metastasis, including MAPK pathway (p-Erk1/2/Erk1/2), JAK-STAT pathway (p-STAT3/STAT3), NF-κB pathway (p-p65/p65), PI3K/AKT pathway (p-AKT/AKT) and EMT markers such as E-cadherin, Vimentin and Fibronectin in MKN45 and AGS cells. P-Erk1/2, p-AKT, p-p65, Vimentin and Fibronectin were downregulated and E-cadherin was upregulated, while p-STAT3 exhibited no significant difference after reduction of Enah in MKN45 and AGS cells (Fig. 7a). To further investigate the role of Enah in EMT in GC, we examined the cell morphology as supplementary. MKN45 and AGS cells after Enah knockdown displayed a round or flat morphology with a short cytoplasmic process compared with control cells, whose morphology was elongated, spindle shaped and scattered (Fig. 7b). We also performed immunofluorescence staining to detect the expression of E-cadherin and Vimentin (Fig. 7c) and the results were consistent with Western blot. Moreover, we measured the expression of related molecules mentioned above, examined the cell morphology and performed immunofluorescence staining after Enah overexpression and got the opposite results (Supplementary Figure 1).

### MAPK (Erk1/2), AKT and NF-κB pathways are involved in enhanced GC cell proliferation and migration induced by Enah overexpression

To verify whether MAPK (Erk1/2), AKT and NF-κB pathways are involved in enhanced GC cell proliferation and migration induced by Enah overexpression, Erk1/2, AKT and NF-κB specific inhibitors GDC-0994(10 mM), PF-04691502(10 mM) and PDTC (100 mM) were used to treat SGC7901-LV-Enah cells at optimal concentration without significant cell death. After treatment with different inhibitors, the corresponding functional assays were performed and the results demonstrated that the abilities of cell proliferation and migration were significantly downregulated in SGC7901-LV-Enah cells after treating with Erk1/2, AKT and NF-κB inhibitors compared with control cells that were treated with DMSO (Supplementary Figure 2).

## Discussion

Even though diagnostic technologies for GC have improved significantly, there is still lack of useful markers for predicting the recurrence and survival of GC patients.

Enah plays an important role in cell adhesion and motility by controlling the geometry of the actin filament network^17,18^. Many reports have demonstrated that Enah was upregulated during the process of malignant transformation of colorectal, cervical, pancreatic and breast cancers^8,12,14,19^. What’s more, Di Modugno et al^12,19^. found that: in breast cancer, Enah overexpression was associated with tumor size, highly invasive properties and advanced clinical stages. In addition, Hu et al^10^. also confirmed that Enah was upregulated in HCC, correlated with tumor differentiation and stage, and most importantly, its overexpression was related to worse prognosis and Enah may be a potential prognostic biomarker for HCC.

In the present study, we have demonstrated that Enah was overexpression in GC tissues and mainly present in the cytoplasm of tumor cells, which were consistent with some previous researches^20–22^. Meanwhile, we measured Enah expression in five GC cell line at mRNA and protein levels. Interestingly, Enah was upregulated in MKN45 and AGS, but not in MKN28, SGC7901, and BGC823, compared with GES. We also found that Enah expression was significantly associated with AJCC stage and depth of invasion. Furthermore, we demonstrated the prognostic significance of Enah in GC, indicating that Enah expression was inversely correlated with OS. Patients with high Enah expression had shorter survival than those with low expression. Multivariate analysis using the Cox proportional hazards model indicated that only depth of invasion, not Enah expression, was an independent prognostic factor for survival in GC patients. Therefore, in some extent, Enah may not be considered as a potential prognostic biomarker for GC patients. As we know, Enah could undergo alternative splicing to produce various protein isoforms with distinct function, which consists of a variant present in primary tumor cells but lost in invasive cells (Mena^11^), two invasive forms (Mena^++^ and Mena^+++^ or Mena^INV^), and the subtype MenaΔv6^23^. And when compared with the 570-amino acids pan-hMena, the isoforms Mena^INV^ (+++) and Mena^++^ contain a supplementary exon, next to the Ena/VASP homology 1 (EVH1) domain (a 4-amino acid region for ++ and a 19-amino acid region for +++); the variant hMena11a contains the exon 11a included within the EVH2 domain, adjacent to the F-actin motif. In contrast, hMenaΔv6 lacks the internal exon 6^23^. Intriguingly, it has been demonstrated that different Mena isoforms could be predictive of patient outcome in mutlivariant analysis^24–28^. Therefore, isoform-specific Enah detection may offer a useful signature for the diagnosis and prognosis of GC.

Next, functional assays in vitro demonstrated that Enah knockdown inhibited cell proliferation, migration, invasion and promoted cell apoptosis in MKN45 and AGS cells, whereas, the overexpression experiment with SGC7901 cell got the opposite results. These results correspond to those of previous studies in other cancers^14,19,29,30^.At the same time, in vivo assay using MKN45 and SGC7901 cells also verified the inhibitory and stimulative role of Enah on cell proliferation and metastasis. Here, we did not perform this assay using the AGS cell because in 2016, R Xing^31^ and colleagues confirmed that the GC cell line AGS, which expresses low level of HLA-I upon activation of the NKp30/MAPK/interleukin (IL)-12 or IL-2 pathway, was susceptible to NK cell-mediated lysis, leading to the failure of AGS cells to form tumors in nude mice. Altogether, our data demonstrate that Enah plays an important role in GC pathogenesis by the promotion of cell proliferation and metastesis and the inhibition of apoptosis.

Furthermore, we confirmed that the activity of MAPK (Erk1/2), AKT pathway and NF-κB pathway were inhibited after Enah silencing while no significant difference was detected in JAK-STAT pathway. Among these three signaling pathways, the former two have been demonstrated that they could be activated by Enah in various tumor cells^14,19,30^, while the latter two were important for cell proliferation and metastasis^32,33^. Besides, we found that E-cadherin, the loss of which contributed to an EMT process and promoted tumor metastasis^34–36^, was upregulated and Vimentin and Fibronectin were downregulated after Enah knockdown, which was also reflected by the change of cell morphology and immunofluorescence staining. At the same time, the opposite results were obtained in GSC7901 cells with Enah overexpression, not only in terms of the change of these three pathways, but also in terms of EMT-related changes. Finally, in order to verify whether MAPK (Erk1/2), AKT, and NF-κB pathways are involved in enhanced GC cell proliferation and migration induced by Enah overexpression, Erk1/2, AKT and NF-κB specific inhibitors were used to treat SGC7901-LV-Enah cells and the results showed that the abilities of cell proliferation and migration were significantly downregulated in SGC7901-LV-Enah cells after treating with Erk1/2, AKT and NF-κB inhibitors. Altogether, these data suggest that MAPK (Erk1/2), AKT, NF-κB pathway and the EMT process may be involved in the role of Enah on GC cell proliferation and metastasis.

Although we investigated the role of Enah on cell proliferation and metastasis with loss-of-function and gain-of-function assays in vitro and in vivo and preliminarily explored the change of signaling pathways and EMT process, the potential molecular mechanism by which Enah exerts on GC needs to be further elucidated in the future. Notably, to our knowledge, EMT is regulated by a complex molecular network including some canonical signaling pathways, such as TGF-β, Wnt and Notch signaling pathways^35^. The Wnt and Notch signalling pathways play important roles in development and tumorigenesis and have reported crosstalks in these processes, which are not well understood^37^. Interestingly, Enah has been demonstrated as a transcriptional target of the Wnt/β-catenin pathway and a novel nexus for the Wnt/β-catenin and the Notch signalling cascades^37^.Moreover, the possible link of Mena with Wnt/β-catenin pathway was also proved experimentally for colorectal, breast and hepatocellular carcinoma cell lines^38^. Besides the well-known role of EMT on tumor cell invasiveness, the link between EMT and angiogenesis was also extensively studied and demonstrated in recent years^39^.Combined with our results, functional Wnt/β-catenin/ Notch-Enah-EMT axis and Enah-EMT-angiogenesis pathway may be good choices for further research.

## Electronic supplementary material

Supplementary Information
