# Author Correction: Enah overexpression is correlated with poor survival and aggressive phenotype in gastric cancer

**DOI:** 10.1038/s41419-019-1573-6

**Published:** 2019-05-07

**Authors:** Di Chen, Li Xu, Xiaowei Li, Yi Chu, Mingzuo Jiang, Bing Xu, Min Zhao, Weijie Wang, Hua Wang, Huijie Kang, Kai Wang, Kaichun Wu, Jie Liang, Gui Ren

**Affiliations:** 1 State Key Laboratory of Cancer Biology and Xijing Hospital of Digestive Diseases, the Air Force Military Medical University, 127 Changle Western Road, Xi’an, Shaanxi Province 710032 China; 2 Department of Hematology, People’s Liberation Army Center of Hematologic Disorders, Xijing Hospital, the Air Force Military Medical University, 127 Changle Western Road, Xi’an, Shaanxi Province 710032 China; 3 Department of Gastroenterology, the 16th Hospital of the People’s Liberation Army of China, 219 Tuanjie South Road, A Letai, Xinjiang Province 836599 China; 4 Department of Gastroenterology, Navy General Hospital, Beijing, 100048 China

**Correction to: Cell Death & Disease 9**, 998 (2018);

10.1038/s41419-018-1031-x, published online 24 September 2018

In the version of this article originally submitted, Fig. 5j included a mismatched image which had been accidentally inserted by the authors during collation of the data. The final data was derived from three independent experiments, and three repetitions for each experiment to ensure the repeatability, reliability and scientificity of the experiment. During this process, the fields overlapped to some extent, giving rise to the mistake. It does not alter any inferences drawn from the data, and the statistical graph is correct. This error has been corrected in both the PDF and HTML versions of the Article.

