# Supplementary material for: Enah overexpression is correlated with poor survival and aggressive phenotype in gastric cancer
Source: Cell Death Dis. 2018 Sep 24;9(10):998. doi: 10.1038/s41419-018-1031-x (PMC6155292; doi:10.1038/s41419-018-1031-x)
Supplement: Supplementary file 1 — Supplementary Information [file 41419_2018_1031_MOESM1_ESM.docx]

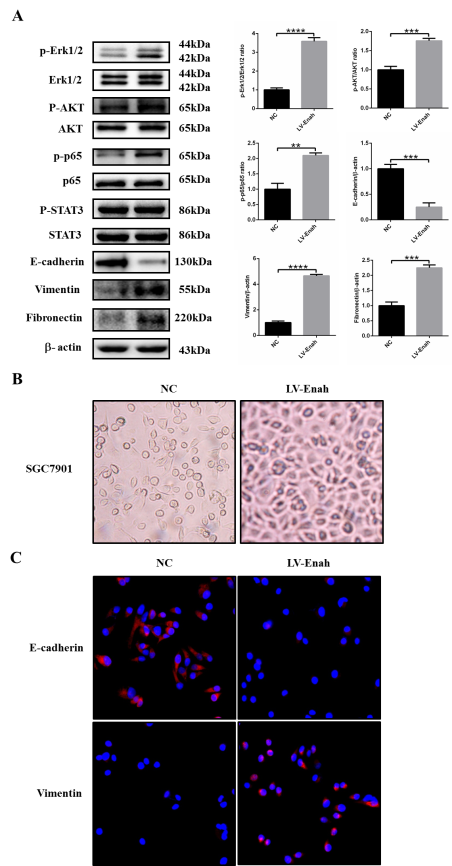


**Supplementary Figure.1. Enah overexpression upregulates p-Erk1/2, p-AKT and p-p65 and promotes EMT progress in GC cell.**

A. Increased protein levels of p-Erk1/2, p-AKT, p-p65, Vimentin, Fibronectin and decreased protein level of E-cadherin in SGC7901-LV-Enah cell compared with NC cell. No significant difference was detected in the protein expression of p-STAT3.

B. Morphology of SGC7901-NC and SGC7901- LV-Enah cells as visualized using phase-contrast microscopy (magnification ×200).

C. Immunofluorescence analysis of E-cadherin and Vimentin expression in SGC7901-NC and SGC7901- LV-Enah cells. **p<0.01, ***p<0.001, ****p<0.0001.


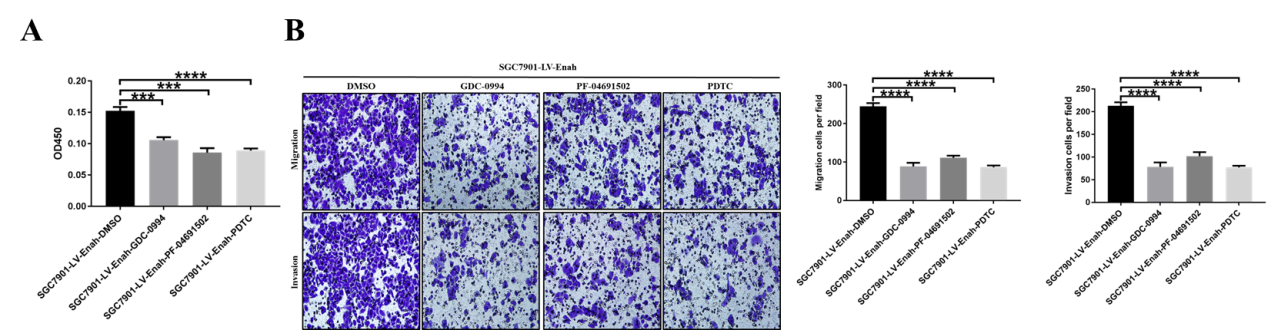


**Supplementary Figure.2. MAPK (Erk1/2), AKT and NF-κB inhibitors reversed enhanced GC cell proliferation and migration induced by Enah overexpression.**

A. BrdU assay showed that less BrdU were incorporated in SGC7901-LV-Enah cells treated with Erk1/2, AKT and NF-κB inhibitors.

B. Transwell assay showed the decreased ability of cell migration and invasion in SGC7901-LV-Enah cells treated with Erk1/2, AKT and NF-κB inhibitors. Representative photographs of migrating and invasive SGC7901 cells, magnification ×200 (left panel) and statistical analysis of the relative numbers of migrating and invasive SGC7901 cells (right panel).

***p<0.001, ****p<0.0001.
